# A Second-Order True-VCO ADC Employing a Digital Pseudo-DCO Suitable for Sensor Arrays

**DOI:** 10.3390/s24248029

**Published:** 2024-12-16

**Authors:** Dante Loi, Victor Medina, Luis Hernandez Corporales

**Affiliations:** Electronics Technology Department, University of Madrid Carlos III, 28911 Leganes, Spain; vmedina@ing.uc3m.es (V.M.); luish@ing.uc3m.es (L.H.C.)

**Keywords:** delta-sigma modulation, true-VCO ADC, pseudo-DCO

## Abstract

This paper explores the implementation of a VCO-based ADC, achieving an ENOB of 12 bits with 1 MHz of a sampling rate in the audio bandwidth. The solution exploits the scalability and PVT invariance of a novel digital-to-frequency converter to reduce the size and consumed power. The architecture has been validated in a 130 nm CMOS technology node displaying a power consumption of 105.57 μW and a silicon footprint of 0.034 mm^2^ in a pseudo-differential configuration. Performance can be dynamically adjusted to trade off power consumption by resolution without changing the sampling rate. In addition, the proposed architecture benefits from multiple instantiations in the same SoC, making it particularly suitable for sensor array applications, such as biomedical sensors and spatial audio arrays.

## 1. Introduction

**Figure 1:**
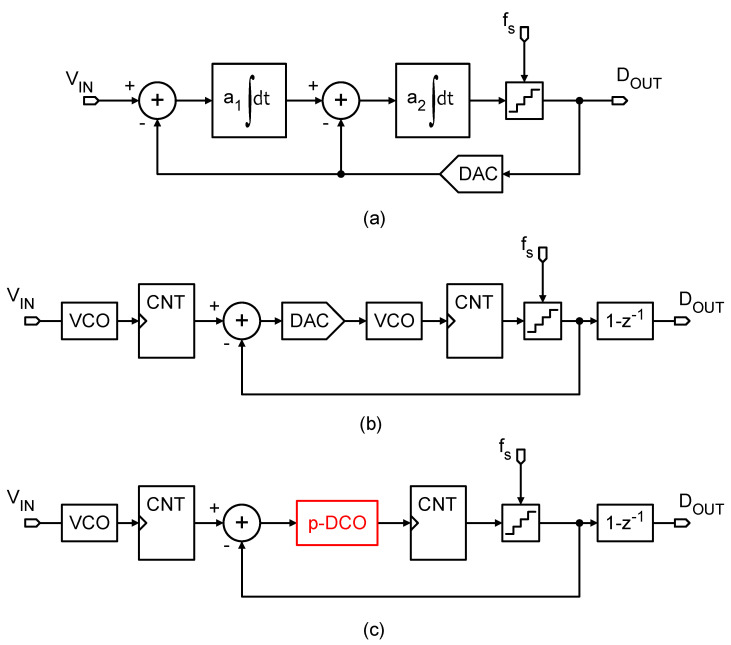
(**a**) Conventional second-order ΔΣ-ADC. (**b**) Equivalent true-VCO ADC requiring a loop DCO. (**c**) Proposed architecture employing a fully digital Pseudo-DCO.

Continuous-time VCO-based ΔΣ ADCs have been widely investigated as an alternative to voltage domain converters [1,2,3], promising better performances and portability to deep sub-micron technology nodes. Despite being a very interesting solution on paper, they have started to be practically adopted only in recent years, due to low intrinsic transistor gain in deep sub-micron processes hindering the performances of conventional implementations. The mostly digital nature of a VCO-based ADC is particularly suitable for low supply voltages where the headroom is limited, and voltage domain solutions would require multistage integrator topologies in order to comply with the gain specifications. In contrast to gm-C integrators, integrators based on pulse frequency modulation (PFM) [4] implemented as a VCO cascaded to an asynchronous accumulator, provide an infinite DC gain at the cost of an additional distortion contribution given by the PFM modulation. However, the contribution of PFM distortion only becomes dominant when the VCO rest frequency is close to the bandwidth of the modulating signal. In virtue of these properties, PFM-integrators are becoming ever more popular as the building block of ΔΣ-ADCs for relatively low-bandwidth applications. In these applications, the VCO rest frequency remains in the MHz range, thus limiting the power share needed by the modulator. A way to further reduce the power of VCO-ADCs is to employ a high-order modulator, for instance, by using the true-VCO architecture [5,6,7]. In [8], a second-order true-VCO ADC for audio applications using binary coding is described. Binary coding allows us to easily enhance the dynamic range of the ADC. Figure 1a depicts the standard second-order ΔΣ-modulator. By replacing the ideal integrators by VCOs and counters, as detailed in [8], we derive the block diagram shown in Figure 1b, which serves as the foundation for this paper. In the architecture proposed in [8], the subtraction is implemented using full-adders and modulo counters, exploiting the properties of modular arithmetic. However, this implementation is susceptible to PVT variations due to the mixed-signal elements in the loop (DAC + VCO). Additionally, it requires trimming to achieve the maximum dynamic range possible. In this brief, we address the aforementioned issues with the system shown in Figure 1b, replacing the asynchronous DAC + VCO with a novel pseudo-digitally controlled oscillator (DCO), as depicted in Figure 1c. The pseudo-DCO was inspired by the digital-to-frequency converter first proposed in [9]. The manuscript is organized as follows: In Section 2, we give a system-level description of the proposed second-order ADC, with particular regard to the pseudo-DCO architecture. Section 3 focuses on transistor-level details and post-layout simulation results. Finally, conclusions are drawn in Section 4.

## 2. System-Level Description of the Proposed Architecture

Direct digital synthesis (DDS) is widely used in many applications as a robust and scalable alternative to mixed-signal digitally controlled oscillators; however, it is not suitable for ultra-low power applications given its quite demanding power requirements. Therefore, we propose a novel topology called pseudo-DCO with the goal of addressing the drawbacks of DDS but maintaining the scalability and robustness to PVT variations.

### 2.1. Pseudo-DCO

**Figure 2:**
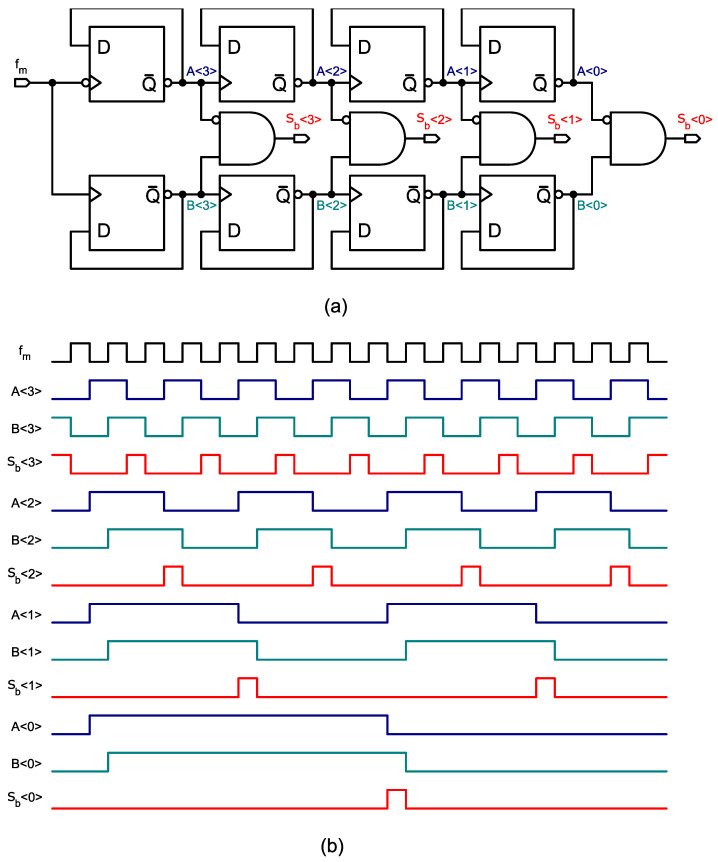
(**a**) Four bits sequence generator the FF must be properly initialize to guarantee the correct functionality of the circuit. (**b**) Relevant sequence generator signals showing the “orthogonality” of the sequences.

**Figure 3:**
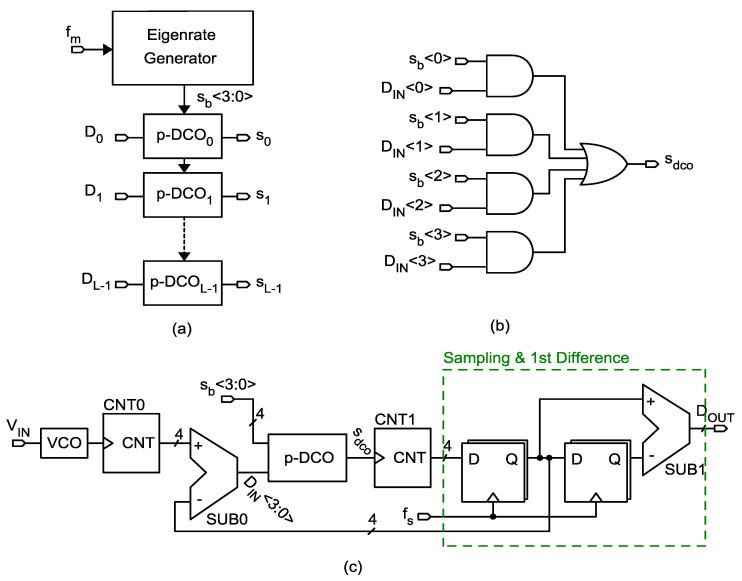
(**a**) Deployment of multiple pseudo-DCOs driven by one sequence generator. (**b**) A 4-bit pseudo-DCO implementation. (**c**) Second-order ΔΣ-ADC implementation.

The pseudo-DCO is based on combining orthogonal signals (eigenrates) generated by a master sequence generator. The sequences of this generator are combined linearly depending on a binary control input. The sequence generator can be shared by several instances of the pseudo-DCO in a SoC (system on a chip) hosting an array of ADCs, as schematically exemplified in Figure 3a. In [9], an early version of pseudo-DCO was described for the implementation of spiking-neural-network hardware accelerators; however, that implementation required delay-lines which are very sensitive to PVT variations and severely compromised the robustness of the system. Figure 2a shows the diagram of a 4-bit sequence generator. The width of the pulses depends on the duty-cycle of the driving clock $f_{m}$. To avoid phase ambiguity, the frequency divider connected D-Flip-Flops must be reset at start-up, which is performed via an asynchronous initialization signal not shown in the figure.

The eigenrate generator produces 4 base sequences $s_{b}$<3:0>, whose frequencies are $f_{m}$ divided by successive powers of two. In the chronograph of Figure 2b, it can be seen that the output pulses of the eigenrates do not overlap. Therefore, they can be combined by means of CMOS OR-gates. The digital-to-frequency conversion is performed selecting the corresponding eigenrates with an AND-gate according to the bits of the digital control word $D_{IN}$. The selected eigenrates are combined in a single-bit signal with a multiple input OR-gate, as shown in Figure 3b. Equation (1) describes the frequency synthesis. On average, the frequency $f_{dco}$ of the output signal $s_{dco}$ is proportional to $D_{IN}$. The frequency-to-input ratio (DCO gain) of the pseudo-DCO is given by $f_{m}/2^{4}$, and its input–output characteristic is expressed in (2).

(1) $$s_{dco}=\sum_{i=0}^{3}D_{IN}<i>\cdot s_{b}<i>$$

(2) $$f_{dco}=\frac{f_{m}}{16}\sum_{i=0}^{3}2^{i}\cdot D_{IN}<i>$$

Combining the eigenrates to generate an arbitrary output frequency inevitably produces a systematic phase error. Although the number of pulses per second will correspond to the desired frequency on average, the output waveform will not have equally spaced pulses. In order to mitigate the effect of such a phase error on the converter performance, it is possible to clock the sequence generator faster (overrunning) and subsequently divide the frequency by means of an asynchronous digital counter in order to maintain the gain of the DCO. This can be practically carried out by increasing the number of flip-flops in CNT1 and only taking the most significant 4 bits as the output. Let ORR represent the over-running ratio of the sequence generator. If we want a pseudo-DCO sensitivity of $f_{s}$, the driving clock must be $f_{m}=2^{4}\cdot \mathrm{ORR}\cdot f_{s}$ in our case. This means we would need an additional $\log_{2}\left(\mathrm{ORR}\right)$ bits in CNT1 (assuming ORR is a power of 2) to properly adjust the frequency.

### 2.2. Input VCO and System-Level Simulations

**Figure 4:**
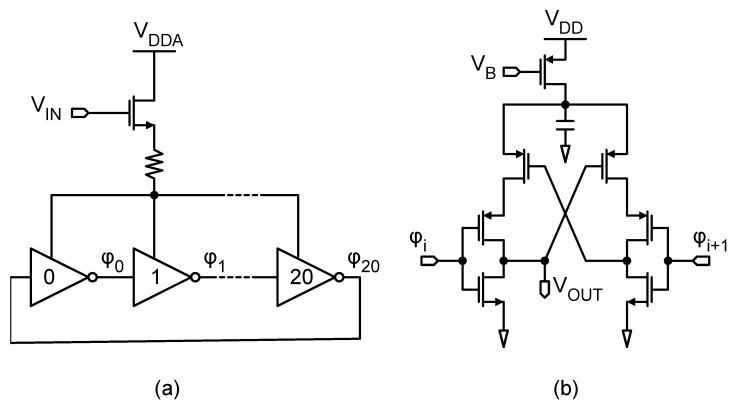
(**a**) Schematic of the 21-tap input VCO. (**b**) Schematic of the level shifter loading the output phases of the VCO.

**Figure 5:**
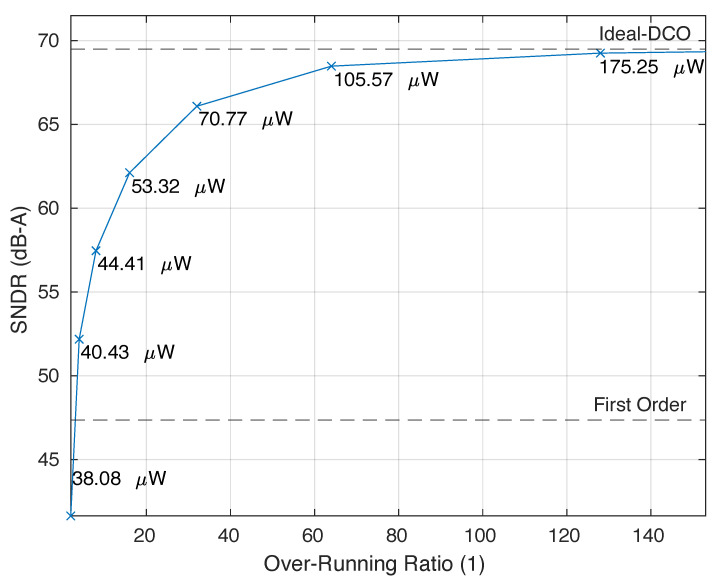
Peak SNDR vs. ORR and post-layout power simulation. The dashed lines are the ideal-DCO and first-order ADC.

The proposed second-order true-VCO ΔΣ-ADC, as shown in Figure 3c, was designed and simulated at both system and transistor levels using a 130 nm CMOS technology. The architecture features a 1 MHz sampling rate and utilizes a 4-bit data-path that includes both input and output counters. The design employs an input VCO implemented as a 21-tap ring oscillator, which is driven by an NMOS transconductor as illustrated in Figure 4a. The resistive degeneration at the source of the transconductor improves the linearity of the VCO, providing negative feedback at the inverters control node. The degeneration resistor was sized to 1 kΩ, and it was implemented as a polysilicon resistor 5 μm wide and 15 μm long. To accurately represent the digital state variables within the 4-bit data-path, the system was initially modeled behaviorally without noise and distortion. These simulations indicated that, for a full-scale input, the system could reach up to 11 output quantization levels without causing saturation of internal state variables, despite an expected maximum of 16 levels. This discrepancy has been previously explained in [8,10]. To maximize the dynamic range of the converter, the rest frequency of the input VCO must be set to 11/2×1 MHz. Assuming an analog supply $V_{DDA}$ = 1.2 V, this is achieved in our design with a DC biasing level of 900 mV at the gate of the input transconductor, yielding a rest frequency of 809 kHz. This frequency is multiplied up to 5.66 MHz by combining 7 phases of the VCO with XOR gates. These seven outputs are equipped with level shifters implemented as in Figure 4b to drive the XOR gates with compatible logic levels, while the rest of the output phases are loaded with dummy-level shifter replicas (see [8]). In the plot of Figure 5, we represent the peak signal-to-noise-and-distortion ratio (SNDR) as the ORR increases. Noticeably, as the performance of the pseudo-DCO asymptotically approaches the ideal-DCO, the asymptotic horizontal line is obtained, replacing the pseudo-DCO with a verilog-A ideal model. In the same figure, the peak SNDR of a first-order ΔΣ-modulator is also reported, and the second-order solution outperforms it for ORR > 3. The dependence of the SNDR on the ORR provides a straightforward mechanism to switch between different power modes. The ORR can be dynamically adjusted depending on the activity of the input signal. For example, in a voice recognition application, the device will most often be in an idle state, only functioning as an activity detector. In this situation, the ORR can drop down to 4, providing an SNDR of 52 dB; however, when the signal of interest is detected, the ORR may ramp up to 64, gaining an additional 16 dB in SNDR. This situation is common in other applications as well, such as biomedical signal acquisition chains.

## 3. Post-Layout Results

**Figure 6:**
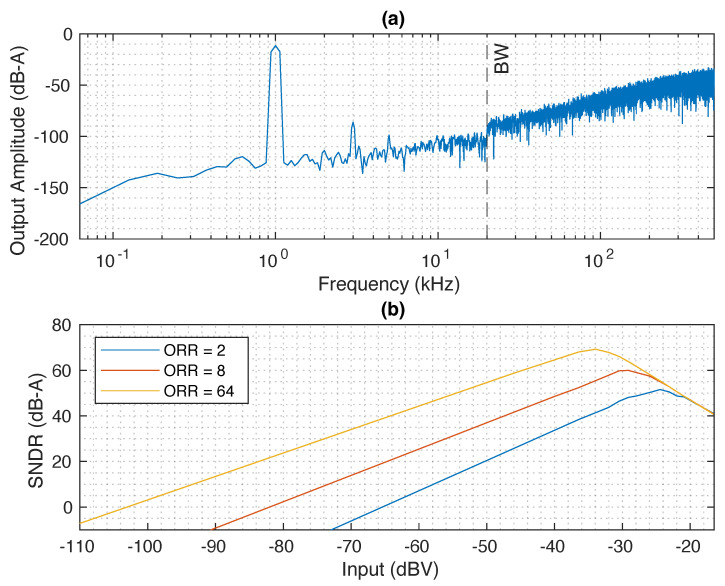
(**a**) Output spectrum of the proposed system with an input of 20 mV. (**b**) Dynamic range of the proposed system, the DR and the peak SNDR scale with the ORR.

**Figure 7:**
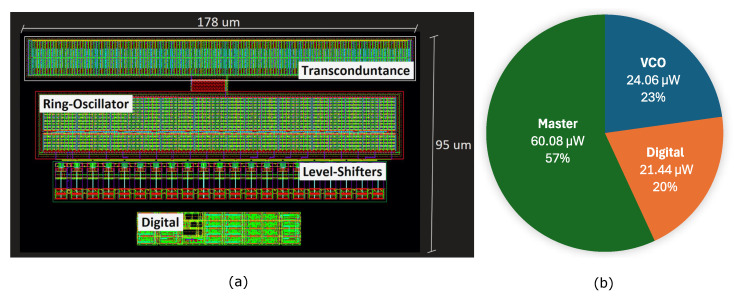
(**a**) Layout of one branch of the proposed converter. (**b**) Power breakdown, with the following parameters: ORR = 64, $f_{s}$ = 1 MHz, $f_{in}$ = 1 kHz, and $A_{in}$ = 20 mV.

The converter in a pseudo-differential configuration [2] was designed and validated with post-layout simulations in a 130 nm CMOS technology node. The ADC core takes 0.034 mm^2^ of a chip area, consuming a total power of 105.57 μW, with an ORR = 64 and a digital supply voltage of 0.9 V. To study the limits of the new frequency-to-digital converter stage, the transconductor and VCO were sized such that their thermal noise and distortion do not limit the SNDR of the converter. According to our calculations, in order to max out the capabilities of the input VCO, we would require a 5-bit data-path. However, the aim of this work is to study the limits of the novel architecture; therefore, we opted for the 4-bit solution. The peak SNDR is 69 dB, which was achieved with a voltage swing of 20 mV around the DC value. In Figure 5, we have annotated some points with the simulated post-layout power consumption of the whole chip to show the power scaling according to the ORR. In Figure 6a, we have represented the averaged output spectrum of the ADC with A-weighted filtering. The input tone is at 1 kHz with an amplitude of 20 mV, which is compatible with the voltage provided by a commercial MEMS microphone. Noticeably, the converter is not limited by the thermal noise floor of the input-VCO, as can be seen from the shaped in-band noise floor. The plot in Figure 6b shows the dynamic range of the converter for a selection of ORR values. All the simulations shown in Figure 6 are performed with a detailed post-layout model, including noise and distortion. Additionally, we did not see any performance degradation across corners, temperature and supply voltage sweeps. The layout of the proposed system is shown in Figure 7a, whilst Figure 7b depicts the power consumption breakdown, from which we can see that the majority of the power is consumed by the eigenrate generator. Table 1 shows a comparison of the proposed circuit with other recently published VCO-based ADCs. The proposed ADC shows the smallest area for the same signal bandwidth. Furthermore, figure of merit (FoM) estimations are in par with other designs. As a further advantage of our solution, the eigenrate generator can be shared among multiple pseudo-DCO instantiations scaling down its power share by the number of pseudo-DCOs in the SoC. This feature makes it particularly suitable for the implementation of ADC arrays. For example, an array of 16 ADCs would reduce the power share of the eigenrate generator per ADC channel to 3.75 μW, making it utterly impactless in the SoC power budget. If 16 ADCs are packed together, the SNDR FoM and DR FoM reported in Table 1 increase to 155 dB, and 176 dB, respectively. The power consumption of the input VCO is independent of the ORR, as it only effects the eigenrate generator and the digital electronics constituting the pseudo-DCO.

## 4. Conclusions

A novel digital-to-frequency converter, referred to as the pseudo-DCO, was proposed and thoroughly characterized. This pseudo-DCO was employed to implement the second stage of a second-order true-VCO ΔΣ modulator in a 130 nm CMOS process. The ADC exhibits excellent performance in post-layout simulations, achieving a peak SNDR of 69 dB and a dynamic range (DR) of 90 dB in maximum performance mode. The device’s performance and power consumption can be dynamically adjusted based on input activity, further improving the FoM. As a predominantly digital design, the converter is less sensitive to PVT variations compared to equivalent mixed-signal implementations, as confirmed by corner and Monte Carlo simulations. Furthermore, the pseudo-DCO scalability property makes it advantageous for multiple instantiations within a single SoC, making it particularly suitable for sensor arrays. Edge devices, where chip area and power consumption are critical constraints, will benefit the most from this proposed solution.

## Figures and Tables

**Table 1:** Comparison with other studies.

Reference	This Work	[8]	[11]	[12]	[13]	[14]
Process	130 nm	130 nm	130 nm	28 nm	65 nm	65 nm
Supply (A/D) (V)	1.2/0.9	1.5/0.95	1.5/0.95	1.1/0.55	1/1	0.6/0.6
BW (kHz)	20	20	20	10	20	100
Area (mm^2^)	0.034	0.095	0.14	0.095	0.11	0.022
Power (uW)	105.6	250	438.1	170.5	142.7	0.094
Sampling (MHz)	1	3.072	3.072	2.4	2	0.2
DR (dB)	90	103	108	91	100.3	N.A.
FOM SNDR (dB)	153	155.5	157	152.6	175.5	176.6
FOM DR (dB)	173	182	184.6	170.4	181.8	N.A.

## Data Availability

Data are available upon request.

## Abbreviations

The following abbreviations are used in this manuscript:

VCO	Voltage controlled oscillator
ADC	Analog-to-digital converter
ENOB	Effective number of bits
PVT	Process, voltage and temperature
CMOS	Complementary metal oxide semiconductor
SoC	System on a chip
DC	Direct current
PFM	Pulse frequency modulation
DAC	Digital-to-analog converter
DDS	Direct digital synthesis
DCO	Digitally controlled oscillator
ORR	Overrunning ratio
SNDR	Signal-to-noise-and-distortion ratio
MEMS	Micro electro-mechanical system
FoM	Figure of merit
